# Cdc42 functions as a regulatory node for tumour‐derived microvesicle biogenesis

**DOI:** 10.1002/jev2.12051

**Published:** 2021-01-12

**Authors:** Jing Wang, Xiangjin Zhuang, Kai Su Greene, Ha Si, Marc A. Antonyak, Joseph E. Druso, Kristin F. Wilson, Richard A. Cerione, Qiyu Feng, Hongyang Wang

**Affiliations:** ^1^ Cancer Research Center The First Affiliated Hospital of USTC Division of Life Sciences and Medicine University of Science and Technology of China Hefei Anhui China; ^2^ National Center for Liver Cancer Eastern Hepatobiliary Surgery Hospital/Institute the Second Military Medical University Shanghai China; ^3^ Department of Molecular Medicine Cornell University Ithaca New York USA; ^4^ Affiliated Hospital of Inner Mongolia University for the Nationalities Tongliao Inner Mongolia China; ^5^ Department of Chemistry and Chemical Biology Cornell University Ithaca New York USA

**Keywords:** Cdc42, EGF signalling, GTPase, IQGAP, Microvesicle, tumour angiogenesis

## Abstract

Tumour‐derived microvesicles (MVs) serve as critical mediators of cell‐to‐cell communication in the tumour microenvironment. So far, the underlying mechanisms of MV biogenesis, especially how key tumorigenesis signals such as abnormal EGF signalling regulates MV release, remain unclear. Here, we set out to establish reliable readouts for MV biogenesis and then explore the molecular mechanisms that regulate MV generation. We found that Rho family small G protein Cdc42 is a convergent node of multiple regulatory signals that occur in MV biogenesis. The binding of activated GTP‐bound Cdc42 and its downstream effector, Ras GTPase‐activating‐like protein 1 (IQGAP1), is required for MV shedding. Activated Cdc42 maintains sustained EGF signalling by inhibiting the internalization of cell surface receptors, including EGFR and the VEGF oligomer, VEGF_90K_, and then facilitates MV release. Subsequently, we further demonstrated that blocking these signalling pathways using the corresponding mutants effectively reduced MV shedding and significantly inhibited MV‐promoted *in vivo* tumour angiogenesis. These findings reveal a complex regulation of MV shedding by tumour cells, shedding light on the regulatory mechanism of MV biogenesis, and potentially contributing to strategies that target MVs in cancer therapy.

## 1 INTRODUCTION

Extracellular vesicles (EVs) are membrane enclosed nano‐ to micron‐sized vesicles that are released from essentially all cell types. They mediate cell‐to‐cell communication by transferring their bioactive cargo consisting of proteins, lipids, and nucleic acids to recipient cells. It is increasingly evident that EVs play a vital role in a variety of physiological or pathophysiological processes (Cocucci et al., 2009; Harding et al., 2013; Raposo & Stoorvogel, 2013; Raposo et al., 1996; Subra et al., 2010; Tkach et al., 2018; Valadi et al., 2007; Willms et al., 2018). The most widely described is their role in cancer (Al‐Nedawi et al., 2009; Costa‐Silva et al., 2015; Desrochers et al., 2016; Fong et al., 2015; Hall et al., 2016; Kalluri, 2016; Lee et al., 2011; Muralidharan‐Chari et al., 2010; Rak, 2010): By regulating intercellular communication, EVs affect the tumour microenvironment, stimulate angiogenesis, and promote tumour growth, thereby contributing to different stages of cancer progression.

Tumour‐derived EVs fall into two broad classes based on their size and generation: Smaller vesicles (small Extracellular Vesicles, small EVs) are mainly exosomes (typically <150 nm in diameter), which are generated from endosomal recycling and multivesicular bodies. Larger vesicles, often commonly referred to as microvesicles (MVs; 200 nm to 2 μm in diameter), which are formed and shed from the plasma membrane (Al‐Nedawi et al., 2009; Cocucci et al., 2009; Desrochers et al., 2016; Hall et al., 2016; Harding et al., 2013; Lee et al., 2011; Raposo & Stoorvogel, 2013; Tkach et al., 2018; Willms et al., 2018). While small EVs are produced in almost all cell types, actin‐based MVs appear to be preferentially produced by tumour cells (Antonyak et al., 2011; Desrochers et al., 2016; Feng et al., 2017; Li et al., 2012). Small EVs have been the most widely studied EV population; however, MVs are likely to play as many critical roles in biology and disease (Harding et al., 2013; Tkach et al., 2018; Willms et al., 2018).  Compared to the smaller small EVs, MVs are capable of carrying more bioactive cargo. When they are released directly from the plasma membrane, cell surface receptors and signalling proteins will remain on the surface of the MVs, which allows them to affect the signalling activities and behaviour of surrounding recipient cells primarily through cell surface signals (Antonyak et al., 2011; Feng et al., 2017). Thus, MVs play a more direct and critical role in the tumour microenvironment than small EVs that are only capable of carrying a very limited amounts of bioactive components. An important early discovery was that MVs shed by glioblastoma cells expressing a mutated form of the EGF receptor, the EGFRvIII, gave rise to enhanced EGFR‐ and oncogenic signalling within neighbouring brain cancer cells (Al‐Nedawi et al., 2008). MVs are capable of conferring enhanced signalling capabilities onto normal fibroblasts, as well as mammary epithelial cells, such that they exhibit the characteristics of transformed cells, including the ability to form tumours in nude mice (Antonyak et al., 2011; Li et al., 2012). Recently, we described how MVs derived from breast cancer cells provide a sustained activation of VEGFRs on endothelial cells *via* a MV‐associated VEGF oligomer, VEGF_90K_, thereby stimulating angiogenesis and promoting tumour growth (Feng et al., 2017).  These findings suggest that MVs provide an important mechanism by which cancer cells at the primary tumour site communicate with their immediate microenvironment (Al‐Nedawi et al., 2008, 2009; Antonyak et al., 2011; Feng et al., 2017; Zomer et al., 2015). Therefore, given the potentially essential role that MVs play in the tumour microenvironment and tumour progression, it is of great interest to understand the nature of the signalling pathway responsible for their biogenesis, especially how key tumorigenesis signals such as abnormal EGF signalling affects MV biogenesis.

The generation and release of MVs are a complicated dynamic process that is regulated by multiple cellular signals.  So far, the underlying mechanisms are still not fully understood.  It is currently known that MV biogenesis does not require the processing, sorting and transport machinery of the classical secretory pathways by which proteins are secreted by trafficking through the endoplasmic reticulum and Golgi.  Actin filaments, which form the basis of cancer cell‐derived MVs (Antonyak et al., 2011), play an important role in their maturation along the cell surface. A few studies involving regulatory mechanisms of MV biogenesis have shown that the Rho family small G protein RhoA, its Rho‐associated coiled‐coil‐containing kinase (ROCK) and Lim kinase signals are required for MV release (Li et al., 2012).  Furthermore, the biogenesis of MVs in cancer cells is also associated with metabolic changes driven by the ‘Warburg effect’, which is thought to be necessary for malignant transformation and cancer progression (Wilson et al., 2013).

In this study, we used MV protein quantification, MV‐associated VEGF_90K_, and the ability of MV to promote tumour angiogenesis as readouts to explore the molecular mechanisms that regulate MV biogenesis. We found that the Rho family small G protein Cdc42 (Etienne‐Manneville & Hall, 2002; Rathinam, 2011; Vega & Ridley, 2008) is pivotal in regulating MV biogenesis. The binding of activated GTP‐bound Cdc42 to its downstream effector, Ras GTPase‐activating‐like protein 1 (IQGAP1) (Fukata et al., 2002; Hedman et al., 2015; Johnson et al., 2009; Noritake, 2005; Watanabe et al., 2004; Watanabe et al., 2005; White et al., 2009), is required for MV shedding. Activated mutants of Cdc42, such as the fast cycling Cdc42F28L mutant (Lin et al., 1997), maintain sustained EGF signalling by blocking EGFR internalization and then help MV release (positive feedback). Activated Cdc42 also promotes MV biogenesis indirectly by inhibiting the internalization of cell surface proteins. Subsequently, we further confirmed that blocking these signalling pathways effectively reduced MV release and significantly inhibited MV‐promoted *in vivo* tumour angiogenesis. We did not examine whether EGF stimulates the release of small EVs. However, the data showed that the shedding of small EVs does not depend on Cdc42. Therefore, these Cdc42‐dependent regulatory mechanisms are unique to MV shedding. These findings provide new insights into the mechanisms regulating MV release by tumour cells, with potential consequences for the design of new therapeutic strategies.

## 2 RESULTS

### 2.1 Isolate and quantify tumour cell‐derived MVs

**FIGURE 1:**
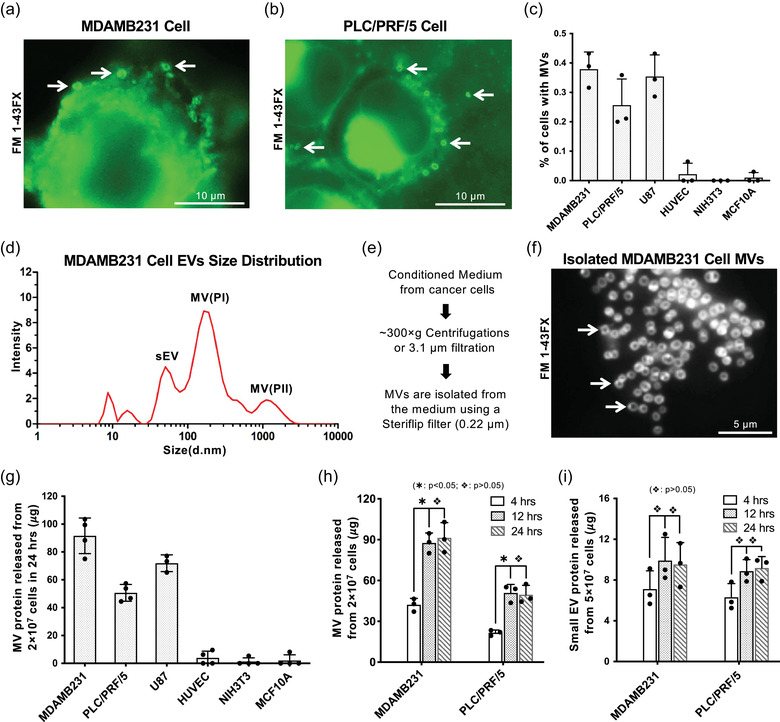
Isolate and quantify tumor cell‐derived MVs. a and b, Serum‐starved MDAMB231 cells (a) or PLC/PRF/5 cells (b) were analyzed by fluorescent staining using the membrane dye FM 1–43FX. Arrows indicate MVs. c, Percentage of different types of human cells having MVs on their surface under serum‐starved condition. MVs were detected by fluorescent staining using the membrane dye FM 1–43FX. d, Particles in conditioned medium collected from MDAMB231 cells were analyzed by dynamic light scattering. The major EV populations were labelled as sEVs (small EVs), MV(PI) (MV population I), and MV(PII) (MV population II). e, Revised MV isolation protocol. f, Size and purity of MVs isolated from MDAMB231 cells were examined by fluorescent staining using the membrane dye FM 1–43FX. Arrows indicate MVs. g, MVs released within 24 h from various cell types cultured under serum‐starved condition were quantified and normalized to the protein amount of MV shed from 2 × 10^7^ cells. h, MVs produced by 2 × 10^7^ MDAMB231 or PLC/PRF/5 cells within 4, 12, or 24 h under serum‐starved condition were quantified. The difference of MV shed within 4 h *vs* 12 or 24 h were statistically significant (∗: *P* < 0.05), and there was no statistical difference between the MV shed within 12 h *vs* 24 h (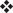:*P* > 0.05). i, Small EVs produced by 5 × 10^7^ MDAMB231 or PLC/PRF/5 cells within 4, 12, or 24 h under serum‐starved condition were quantified. There was no statistical difference between the small EVs secreted within 12 and 24 h (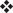:*P* > 0.05)

**FIGURE 2:**
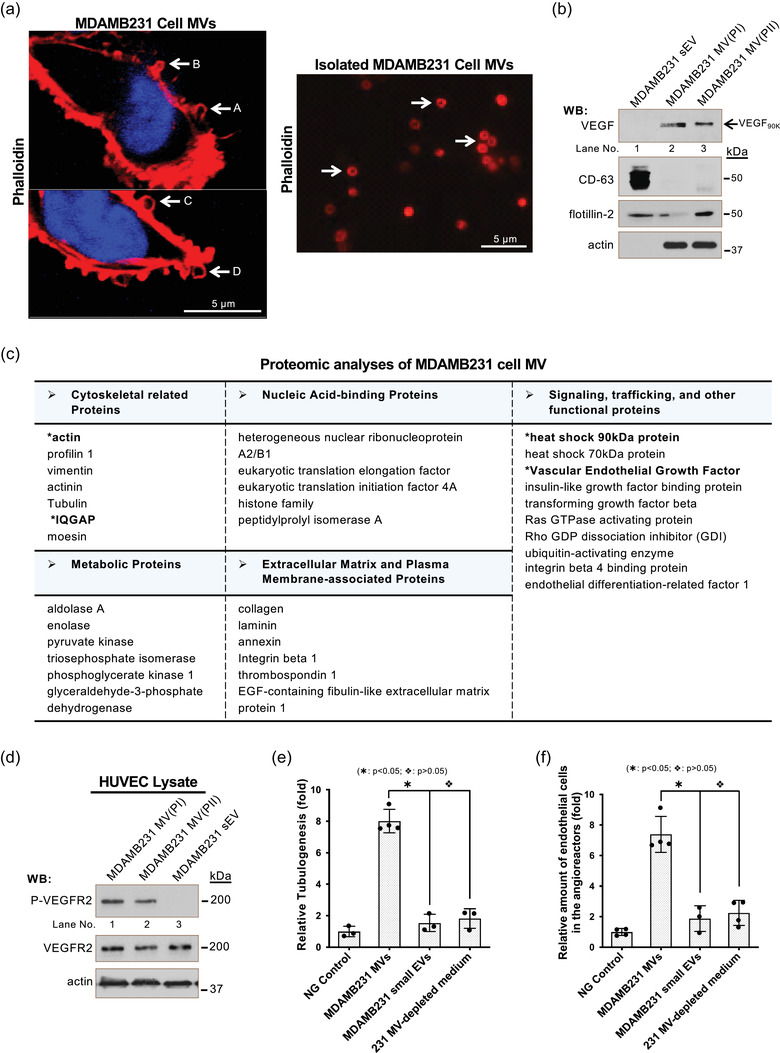
VEGF_90K_ and angiogenesis are biomarker and functional readouts of MV. a, MDAMB231 cells were permeabilized and analyzed by confocal fluorescent microscopy using Rhodamine‐conjugated phalloidin to detect F‐actin (left panel). Arrows indicate the MVs at different steps of shedding: budding (arrow A), stretching (arrow B), closing (arrow C), and release (arrow D). MVs isolated from MDAMB231 cells were examined by fluorescent staining using Rhodamine‐conjugated phalloidin (right panel). Arrows indicate MVs. b, Small EVs (lane 1) or MVs (MV(PI): lane 2 and MV(PII): lane 3) shed from MDAMB231 cells were isolated, lysed, and immunoblotted (5 μg/samples) with antibodies against VEGF, flotillin‐2, actin, and the small EV marker CD‐63 (Logozzi et al., 2009). c, Proteomic analysis of MDAMB231 cell MVs isolated using the revised procedure (Figure 1e) was carried out and protein identified was compiled based on general cellular function. d, Lysates (15μg/samples) of serum‐deprived HUVECs exposed to MV(PI) (lane 1), MV(PII) (lane 2), or small EVs (lane 3) from MDAMB231 cells (5 μg/ml of small EV or MV proteins) for 15 min were immunoblotted with antibodies against phosphorylated VEGFR2 (P‐VEGFR2), total VEGFR2, or actin. e, Tubulogenesis assays were carried out on HUVECs treated with serum‐free RPMI medium (Negative (NG) control; histogram 1), MVs (10 μg protein/ml, histogram 2) or small EVs (10 μg protein/ml, histogram 3) from MDAMB231 cells, or MV‐depleted conditioned medium from MDAMB231 cell culture (10 μg protein/ml, histogram 4). The relative differences in tube lengths were plotted (histogram 2 *vs* histograms 3 or 4; 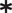: *P* < 0.05, histogram 3 *vs* histogram 4; 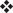: *P* > 0.05). f, The relative amounts of endothelial cells that entered the implanted angioreactors that lacked activators (vehicle only, Negative (NG) control; histogram 1), contained MDAMB231 cell MVs (2 μg total protein/angioreactor), MDAMB231 cell small EVs (2 μg total protein/angioreactor), or MV‐depleted conditioned medium from MDAMB231 cell culture (2 μg total protein/angioreactor). The relative amounts of endothelial cells that entered the implanted angioreactors were plotted (histogram 2 *vs* histograms 3 or 4; 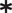: *P* < 0.05, histogram 3 *vs* histogram 4; 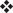: *P* > 0.05)

Tumour cell‐derived MVs are membrane enclosed vesicles that bud and are released from the plasma membrane (Cocucci et al., 2009; Lee et al., 2011; Raposo & Stoorvogel, 2013; Willms et al., 2018). They can be visualized by scanning electron microscopy (SEM) (Figure 1A in ref. Antonyak et al., 2011; Figure 2d in ref. Feng et al., 2017) or by fluorescent microscopy (Figures 1A, 1C, and 1D in ref. Antonyak et al., 2011; Figure 2e, 2f, and 3c in ref. Feng et al., 2017) (Antonyak et al., 2011; Feng et al., 2017). Analysis of serum‐starved culture of cancer cell lines, such as MDAMB231 (Figure 1a) and PLC/PRF/P5 cells (Figure 1b), by fluorescent microscopy after staining with the lipid‐binding membrane dye, FM1‐43X, showed that these MVs are either still present on cell surfaces or have been released into medium (Figure 1A and 1b, arrows).  For the cancer cell lines we examined, the percentage of the cancer cells with distinct MVs on their surfaces (presumably indicating MV shedding ability) ranges from ∼20% to ∼45% (Figure 1c). Conversely, no MVs were detected on the surfaces of normal (non‐transformed) cells such as NIH3T3 fibroblasts, HUVEC endothelial cells, and MCF10A epithelial cells, indicating that MV may be preferentially produced by cancer cells.

**FIGURE 3:**
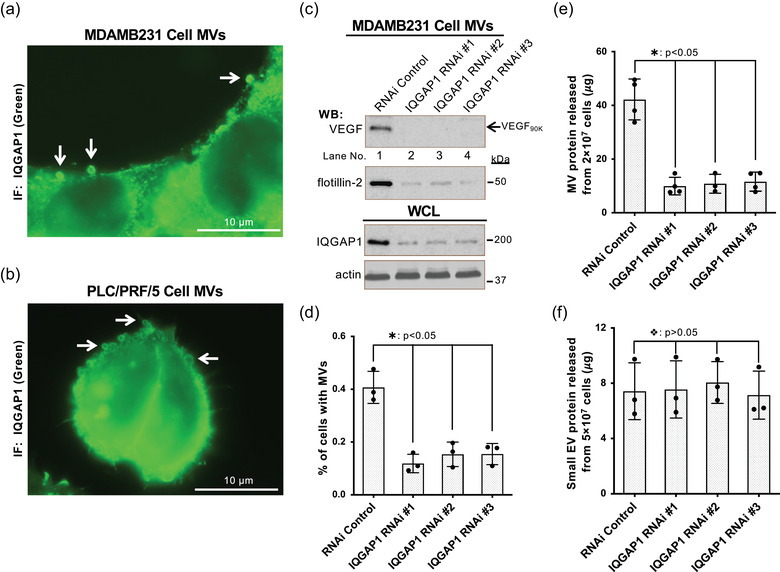
IQGAP1 is necessary for MV biogenesis. a and b, Serum‐starved MDAMB231 (a) or PLC/PRF/5 (b) cells were analyzed by immunofluorescent microscopy using IQGAP1 antibody. Arrows indicate the IQGAP1 localized on MVs. c, MVs shed within 4 h from the same number of MDAMB231 cells transfected with control RNAi (lane 1) or RNAis targeting IQGAP1 (lanes 2–4) were immunoblotted with antibodies against VEGF and flotillin‐2, while the whole cell lysates (WCLs, 15 μg/samples) were immunoblotted with antibodies against IQGAP1 and actin. d, Percentage of MDAMB231 cells transfected with control RNAi (histogram 1) or RNAis targeting IQGAP1 (histograms 2–4) having MVs on their surface under serum‐starved condition. MVs were detected by fluorescent staining using Rhodamine‐conjugated phalloidin. The difference of the percentage of these cells having MV was statistically significant (histogram 1 *vs* histograms 2–4; 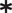: *P* < 0.05). e‐f, MVs (e) or small EVs (f) produced within 4 h by the same number of MDAMB231 cells transfected with control RNAi (histogram 1) or RNAis targeting IQGAP1 (histograms 2–4) were quantified. The difference of the MVs shed from these cells were statistically significant (histogram 1 *vs* histograms 2–4; 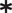: *P* < 0.05) (e). The difference of the small EVs secreted from these cells were not statistically significant (histogram 1 *vs* histograms 2–4; 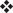: *P* > 0.05) (f)

To examine the size distribution of MV and other EV populations,  we analyzed conditioned medium of MDAMB231 cells by dynamic light scattering (DLS) (Santana et al., 2014) after low speed centrifugation (∼300 × *g*). EVs derived from MDAMB231 cells can be categorized into three populations based on their sizes, small EVs (30‐150 nm in diameter, labelled as sEV), smaller MVs (100‐350 nm in diameter, labelled as MV population I, MV(PI)), and larger MVs (0.5‐2 um in diameter, labelled as MV population II, MV(PII)) (Figure 1d). The size of MV(PII) measured by dynamic light scattering coincides with the size of the MVs observed by fluorescent microscopy (Figure 1a). Because the larger MV(PII) population tends to be ‘lost’ (precipitated into pellet) when higher sedimentation forces (>3000 × *g*, Figures S1A and S1B) are used during MV isolation, we adopted low‐speed centrifugation or filtration with a 3.1 μm filter to ensure the capture of the MV(PII) population (Figure 1e). The isolated MV preparation was examined by fluorescent microscopy to verify the presence of MV(PII) (Figure 1f).

Quantification of the MV preparation isolated by the revised procedure (Figure 1e) showed that 2 × 10^7^ cancer cells could release ∼40 to ∼120μg MV protein within 24 h (Figure 1g). MV(PI) and MV(PII) populations are isolated with revised protocol (Figure S1C) and MV(PII) accounted for more than 80% of total MV protein (Figure S1E). The majority of previous MV studies allow release of MV for 24 h or even longer before collection, which could lead to saturation of MVs in the medium and distort MV protein quantification. To determine the best timing, we measured MV protein released by MDAMB231 or PLC/PRF/P5 cells in 4, 12 and 24 h and found that MV shedding indeed saturates in the culturing medium in about 12 h (Figure 1h). Therefore, in subsequent studies, we collect EVs released from 2 × 10^7^ cancer cells within 4 h for more accurate quantification unless otherwise specified. We also found that small EVs carried much less protein cargo than MVs (Figures S1D and S1F) and tend to saturate in conditioned medium faster than MVs (Figure 1i).

### 2.2 VEGF_90K_ and angiogenesis are reliable biomarkers and functional readouts for MV

Appropriate biomarkers and functional readouts are necessary for studying MV biogenesis. The biogenesis of MVs is a complicated process tightly related to actin cytoskeletal rearrangements, and multiple steps in this process, such as budding (Figure 2a, arrow A), stretching (Figure 2a, arrow B), closing (Figure 2a, arrow C), and release (Figure 2a, arrow D), can be visualized by fluorescent staining with rhodamine‐conjugated phalloidin. Immunoblot analysis indicated that actin is enriched within the two MV populations and does not exist in small EVs (Figure 2b). However, actin is an abundant protein that also present in other cellular fractions, as well as within cell debris, which makes it a less suitable MV‐specific biomarker. Cell membrane proteins, such as flotillin‐2, are commonly used as MV or EV markers. Unfortunately, they are usually not specific for MVs. For example, flotillin 2 is also present in small EVs (Figure 2b).

To determine appropriate MV‐specific biomarkers and functional readouts for MVs, we carried out proteomic analysis of MDAMB231 cell‐derived MVs, isolated using the revised procedure (Figure 1e). Identified proteins were compiled based on their general cellular functions (Antonyak et al., 2011) (Figure 2c).  Notably, among the MV‐associated proteins were the cytoskeleton related proteins, actin and IQGAP1, and signalling proteins, such as VEGF and Hsp90. In previous studies (Feng et al., 2017), we found that the VEGF oligomer, VEGF_90K_, only presents on MVs, but not on small EVs (Figure 3d, ref. Feng et al., 2017; Figure 2b) and confers MV‐specific cellular functions.  As previously shown (Feng et al., 2017) (also see Figure 2d), MV‐associated VEGF_90K_ activated VEGFR2 and stimulated tubulogenesis of HUVECs in an *in vitro* angiogenesis (Figure 2e) (Al‐Nedawi et al., 2008, 2009; Arnaoutova & Kleinman, 2010; Feng et al., 2017). In contrast, small EVs failed to activate VEGFR2 (Figure 2d), and neither small EVs nor MV‐depleted conditioned medium (soluble peptides and small EVs) stimulated tubulogenesis (Figure 2e and Figure S2A). We then employed subcutaneously implanted angioreactors (Figure S2B) (Feng et al., 2017; Napoli et al., 2011) to examine the ability of MV, small EVs, and MV‐depleted conditioned medium to stimulate angiogenesis *in vivo*.  Only MDAMB231 cell‐derived MVs were able to effectively stimulate the migration of endothelial cells into angioreactors (Figure 2f and Figure S2B). Collectively, these data suggest that VEGF_90K_‐associated MVs, but not small EVs or soluble peptides in conditioned medium, are capable of stimulating angiogenesis. The unique presence of VEGF_90K_ on MV makes it a valuable biomarker of MV and also allows the use of angiogenesis as a functional readout when studying MV biogenesis.

### 2.3 IQGAP1 is necessary for MV biogenesis

Proteomic screening of MDAMB231 cell‐derived MVs identified IQGAP1 (Figure 2c), a scaffold protein that integrates signals regulating various cellular processes ranging from organization of the actin cytoskeleton to cell cycle. IQGAP1 is of particular interest as a therapeutic target because it serves as a node for many signalling pathways implicated in cancer progression (Hedman et al., 2015; Johnson et al., 2009; Smith et al., 2015; White et al., 2009). It is well known that IQGAP1 associates with cytoskeletal proteins, particularly actin, and regulates actin cytoskeletal rearrangement (Choi & Anderson, 2016; Noritake, 2005; Smith et al., 2015; Watanabe et al., 2015).  Therefore, IQGAP1 has a potential functional role in the biogenesis of actin‐based MVs.

By immunoblot analysis (Figure S3A) and immunofluorescent (IF) staining with an IQGAP1 antibody, we confirmed that IQGAP1 is a component of MVs derived from MDAMB231 (Figure 3a) or PLC/PRF/P5 cells (Figure 3b). To determine how IQGAP1 affects MV biogenesis, we knocked down IQGAP1 expression in MDAMB231 cells using RNAi. Knockdown of IQGAP1 (Figure 3c) significantly reduced the percentage of cells having MVs on their surface (Figure 3d). Quantification of MVs shed from 2 × 10^7^ cancer cells within 4 h showed that the RNAi‐mediated knockdowns of IQGAP1 in MDAMB231 cells dramatically inhibited MV shedding (Figure 3e). Moreover, VEGF_90K_ and flotillin‐2 were found to be significantly reduced in the MV preparations after IQGAP1 knockdown (Figure 3c). In contrast, small EV secretion was not affected by the knockdown of IQGAP1 (Figure 3f). These data suggest that IQGAP1 is required for the release of MVs, but not small EVs.

From here on, in order to examine the biogenesis of MV, unless specified in the figure legend, MVs shed from the same number of cells were used in MV protein quantification, western blotting (such as Figure 3c), tubulogenesis assays and *in vivo* angiogenesis assays.

### 2.4 Rho family GTPase Cdc42 regulates MV biogenesis

Cdc42, a member of the Rho family of GTP‐binding proteins, functions as a molecular switch in a wide variety of cellular responses (Etienne‐Manneville & Hall, 2002; Rathinam, 2011; Vega & Ridley, 2008). As a binding partner and activator of IQGAP1, Cdc42, together with IQGAP1, is involved in the regulation of the actin cytoskeletal architecture (Hedman et al., 2015; Johnson et al., 2009; Watanabe et al., 2015; White et al., 2009). Since IQGAP1 is required for the biogenesis of actin‐based MVs, we wondered whether Cdc42 is also necessary for MV generation.

**FIGURE 4:**
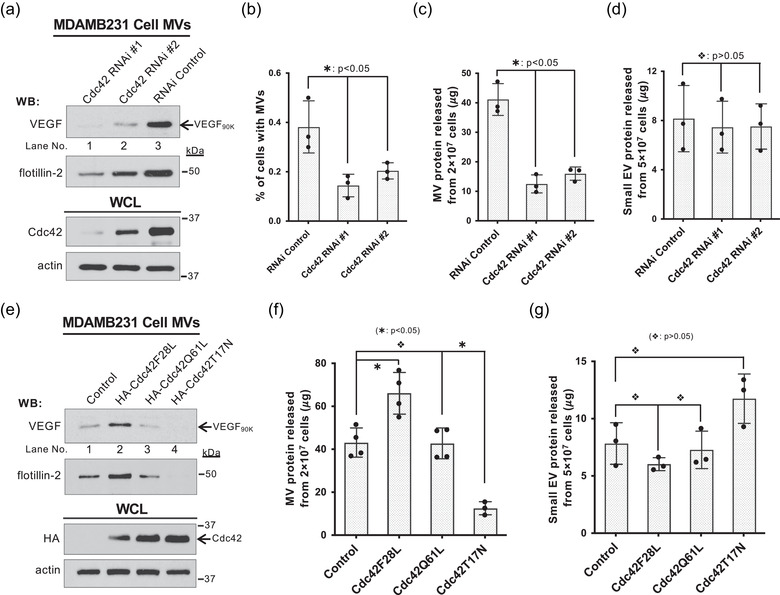
Rho family GTPase Cdc42 regulates MV biogenesis. a, MVs shed within 4 h from the same number of MDAMB231 cells transfected with control RNAi (lane 3) or RNAis targeting Cdc42 (lanes 1–2) were immunoblotted with antibodies against VEGF and flotillin‐2, while the whole cell lysates (15 μg/samples) were immunoblotted with antibodies against Cdc42 and actin. b, Percentage of MDAMB231 cells transfected with control siRNA (histogram 1) or RNAis targeting Cdc42 (histograms 2 and 3) having MVs on their surface under serum‐starved condition. MVs were detected by fluorescent staining using Rhodamine‐conjugated phalloidin. The difference of the percentage of these cells having MV was statistically significant (histogram 1 *vs* histograms 2 or 3; 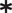: *P* < 0.05). c‐d, MVs (c) or small EVs (d) produced within 4 h by the same number of MDAMB231 cells transfected with control RNAi (histogram 1) or RNAis targeting Cdc42 (histograms 2 and 3) were quantified. The difference of the MV shed from these cells were statistically significant (histogram 1 *vs* histograms 2 or 3; 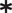: *P* < 0.05) (c). The difference of the small EVs secreted from these cells were not statistically significant (histogram 1 *vs* histograms 2 or 3; 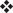: *P* > 0.05) (d). e, MVs shed within 4 h from the same number of MDAMB231 cells expressing empty vector (control, lane 1), HA‐tagged Cdc42F28L (lane 2), HA‐tagged Cdc42Q61L (lane 3), or HA‐tagged Cdc42T17N (lane 4) were immunoblotted with antibodies against VEGF and flotillin‐2, while the whole cell lysates (15 μg/samples) were immunoblotted with antibodies against HA and actin. f‐g, MVs (f) or small EVs (g) produced within 4 h by the same number of MDAMB231 cells expressing empty vector (control, histogram 1), HA‐tagged Cdc42F28L (histogram 2), HA‐tagged Cdc42Q61L (histogram 3), or HA‐tagged Cdc42T17N (histogram 4) were quantified. Cdc42F28L promoted MV release (histogram 2 *vs* histogram 1; 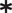: *P* < 0.05), Cdc42Q61L had no significant effect on MV production (histogram 3 *vs* histogram 1; 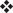: *P* > 0.05), while Cdc42T17N inhibited MV biogenesis (histogram 4 *vs* histogram 1; 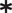: *P* < 0.05) (f). Cdc42 mutants had no effect on small EV secretion (histogram 1 *vs* histograms 2–4; 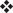: *P* > 0.05) (g)

MVs shed from MDAMB231 cells in which Cdc42 was knocked down by RNAi were isolated and quantified (Figure 4a). These Cdc42‐knockdowned MDAMB231 cells showed a significant decrease in the percentage of cells with MVs on their surfaces (Figure 4b). Both MV protein quantification (Figure 4c) and immunoblot detecting VEGF_90K_ and flotillin‐2 (Figure 4a) indicated that the RNAi‐mediated knockdown of Cdc42 in MDAMB231 cells dramatically inhibited MV shedding, suggesting that Cdc42 is indeed required for MV biogenesis. In contrast, the small EV secretion was not affected by the knockdown of Cdc42 (Figure 4d).

We further examined the role of Cdc42 in regulating MV biogenesis by expressing several well‐established Cdc42 mutants (Etienne‐Manneville & Hall, 2002; Li et al., 1999; Lin et al., 1997; Rathinam, 2011; Vega & Ridley, 2008; Wu et al., 2003). The Cdc42(Q61L) mutant (Labelled as Cdc42Q61L hereafter) is GTPase defective and locked in the GTP‐bound state. Cdc42(F28L) (Labelled as Cdc42F28L hereafter) is an activated ‘fast‐cycling’ Cdc42 mutant that is capable of constitutive GDP‐GTP exchange (Lin et al., 1997). Cdc42(T17N) (Labelled as Cdc42T17N hereafter) is a dominant negative Cdc42 mutant. MVs shed from the same number of MDAMB231 cells expressing Cdc42Q61L, Cdc42F28L, or Cdc42T17N were quantified and detected by immunoblotting for the presence of VEGF_90K_ and flotillin‐2. As shown in Figures 4E and 4f, Cdc42F28L promoted MV release, Cdc42Q61L had no significant effect on MV production, whereas Cdc42T17N inhibited MV biogenesis. These results indicate that Cdc42 activity is necessary for MV biogenesis and the GDP‐GTP exchange activity of Cdc42 facilitates MV shedding. In contrast, small EV secretion was not significantly affected by the expression of these Cdc42 mutants (Figure 4g), which further confirmed that small EV secretion and MV biogenesis are regulated by different signalling pathways.

### 2.5 Cdc42‐IQGAP signalling is required for MV biogenesis

**FIGURE 5:**
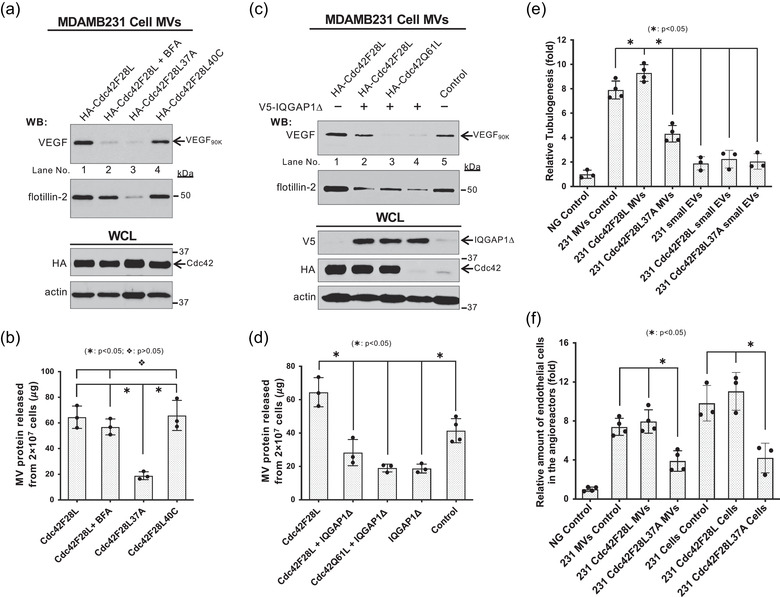
Cdc42‐IQGAP signaling is required for MV biogenesis. a, MVs shed within 4 h from the same number of MDAMB231 cells expressing HA‐tagged Cdc42F28L without (lane 1) or with BFA treatment (lane 2), HA‐tagged Cdc42F28L37A (lane 3), or HA‐tagged Cdc42F28L40C (lane 4) were immunoblotted with antibodies against VEGF and flotillin‐2, while the whole cell lysates (15 μg/samples) were immunoblotted with antibodies against HA and actin. b, MVs released within 4 h from 2 × 10^7^ MDAMB231 cells expressing HA‐tagged Cdc42F28L without (histogram 1) or with BFA treatment (histogram 2), HA‐tagged Cdc42F28L37A (histogram 3), or HA‐tagged Cdc42F28L40C (histogram 4) were isolated and quantified. The difference of MV shed from the same number of MDAMB231 cells expressing Cdc42F28L37A mutant and other MV preparations were statistically significant (histogram 3 *vs* histograms 1, 2, or 4; 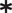: *P* < 0.05). The difference of MV shed from the same number of MDAMB231 cells expressing Cdc42F28L40C mutant and Cdc42F28L without or with BFA treatment were not statistically significant (histogram 4 *vs* histograms 1 or 2; 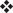: *P* > 0.05). c, MVs shed within 4 h from the same number of MDAMB231 cells expressing HA‐tagged Cdc42F28L alone (lane 1) or together with V5‐tagged IQGAPΔ (lane 2), HA‐tagged Cdc42Q61L together with V5‐tagged IQGAPΔ (lane 3), V5‐tagged IQGAPΔ alone (lane 4), or empty vector (control, lane 5) were immunoblotted with antibodies against VEGF and flotillin‐2, while the whole cell lysates (15 μg/samples) were immunoblotted with antibodies against HA, V5, and actin. d, MVs released within 4 h from 2 × 10^7^ MDAMB231 cells expressing HA‐tagged Cdc42F28L alone (histogram 1) or together with V5‐tagged IQGAPΔ (histogram 2), HA‐tagged Cdc42Q61L together with V5‐tagged IQGAPΔ (histogram 3), V5‐tagged IQGAPΔ alone (histogram 4), or empty vector (histogram 5) were isolated and quantified. The difference of MV shed from the same number of MDAMB231 cells expressing IQGAPΔ mutant and other MV preparations were statistically significant (histograms 2–4 *vs* histogram 1; 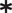: *P* < 0.05, histograms 2–4 *vs* histogram 5; 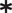: *P* < 0.05). e, Tubulogenesis assays were carried out on HUVECs treated with serum‐free RPMI medium (Negative (NG) control; histogram 1), MVs (histograms 2–4) or small EVs (histograms 5–7) from MDAMB231 cells transfected with empty vector (histograms 2 and 5), HA‐tagged Cdc42F28L (histograms 3 and 6), HA‐tagged Cdc42F28L37A (histograms 4 and 7). 10 μg/ml protein were used in MV or small EV control (histograms 2 and 5). Other MV or small EV preparations were normalized to the protein amount of MVs or small EVs shed from the same number of MDAMB231 cells (compared to MV or small EV control). The relative differences in tube lengths were plotted (histogram 2 *vs* histograms 4–7; 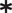: *P* < 0.05, histogram 4 *vs* histograms 2 or 3; 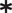: *P* < 0.05). f, The relative amounts of endothelial cells that entered the implanted angioreactors that lacked activators (vehicle only, Negative (NG) control, histogram 1), contained MDAMB231 cells (5 × 10^4^ cells/angioreactor) transfected with empty vector (histogram 5), HA‐tagged Cdc42F28L (histogram 6), HA‐tagged Cdc42F28L37A (histogram 7), or MV shed from these various MDAMB231 cells (histograms 2–4). 2 μg total protein/angioreactor was for MV control (histogram 2). Other MV preparations (histograms 3 and 4) were normalized to the protein amount of MVs shed from the same number of MDAMB231 cells (compared to MV control). The relative amounts of endothelial cells that entered the implanted angioreactors were plotted (histogram 4 *vs* histograms 2 or 3; 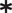: *P* < 0.05, histogram 7 *vs* histograms 5 or 6; 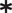: *P* < 0.05)

As a key molecular switch, Cdc42 activates a variety of downstream effectors, including IQGAP1, WASP, PAK and several other signalling targets. (Etienne‐Manneville & Hall, 2002; Johnson, 1999; Li et al., 1999; Rathinam, 2011; Vega & Ridley, 2008). In addition to Cdc42, IQGAP1 can also be activated by other Rho family GTPases, such as Rac. To determine whether Cdc42 regulates MV biogenesis by activating IQGAP, we examined MV shedding upon overexpression of two Cdc42 double mutants, Cdc42(F28L,F37A) (Labelled as Cdc42F28L37A hereafter) and Cdc42(F28L,Y40C) (Labelled as Cdc42F28L40C hereafter) (Johnson, 1999; Li et al., 1999). Cdc42F28L37A is a constitutively active, IQGAP binding‐defective double mutant (Figure S5A) which still binds to other Cdc42 effectors such as PAK and WASP. In contrast, the Cdc42F28L40C double mutant binds to IQGAP1, but does not associate with PAK and WASP. MVs shed from the same number of MDAMB231 cells expressing HA‐tagged Cdc42F28L, Cdc42F28L37A or Cdc42F28L40C were isolated and quantified. Expression of Cdc42F28L37A, but not Cdc42F28L40C, blocked MV shedding (Figure 5a, lane 3 *vs* lane 4; Figure 5b, histogram 3 *vs* histogram 4), suggesting that Cdc42 regulates MV biogenesis directly through IQGAP1. While the Golgi‐dependent conventional secretion inhibitor brefeldin A (BFA) (Feng et al., 2003) reduced the amount of VEGF_90K_ on MVs derived from MDAMB231 cells expressing Cdc42F28L (Figure 5a, top panel, lane 1 *vs* lane 2), it did not change the total amount of released MVs (Figure 2, 5, 2nd panel from top, lane 2; Figure 5b), suggesting that MV biogenesis is independent of the traditional secretory pathways and its shedding is not affected by cargo loading.

To further confirm the regulatory role of Cdc42‐IQGAP1 signalling in MV shedding, we expressed V5‐tagged IQGAP1Δ, a Cdc42 binding‐defective mutant (Rittmeyer et al., 2008; Wang et al., 2009) (IQGAP1‐FΔMK in ref. Rittmeyer et al., 2008; Figure S4A), together with Cdc42F28L, Cdc42Q61L, or empty vector in MDAMB231 cells. Expression of the IQGAP1Δ mutant dramatically decreased MV shedding even when co‐expressing it with Cdc42F28L, as demonstrated by reduced VEGF_90K_ and flotillin‐2, as well as by MV protein quantification (Figure 5c, lanes 2–4; Figure 5d). The results establish that IQGAP1 is required for the regulation of MV shedding by Cdc42 and that the shedding process is not dependent on the Golgi‐dependent conventional secretory pathway.

Next, we examined the role of Cdc42‐IQGAP1 signalling in MV biogenesis through functional analysis. Intact MVs or small EVs released from the same number of MDAMB231 cell expressing empty vector, Cdc42F28L, or Cdc42F28L37A were isolated and examined for their ability to stimulate angiogenesis *in vitro* by the tubulogenesis assays as described above (Al‐Nedawi et al., 2008, 2009; Arnaoutova & Kleinman, 2010; Feng et al., 2017). As shown in Figure 5e, MVs, but not small EVs, stimulated angiogenesis *in vitro*, and the Cdc42F28L37A mutant impaired the ability of MVs to stimulate angiogenesis (Figures 5e and S4B). In the *in vivo* angiogenesis assays, expression of Cdc42F28L37A significantly reduced the ability of MDAMB231 cells and intact MVs derived from these cells to stimulate the migration of endothelial cells into the angioreactors (Figures 5f and S4C). These data, taken together with the above results, confirm that Cdc42‐IQGAP1 signalling regulates MV shedding and function.

### 2.6 Sustained EGF signalling facilitates MV biogenesis

**FIGURE 6:**
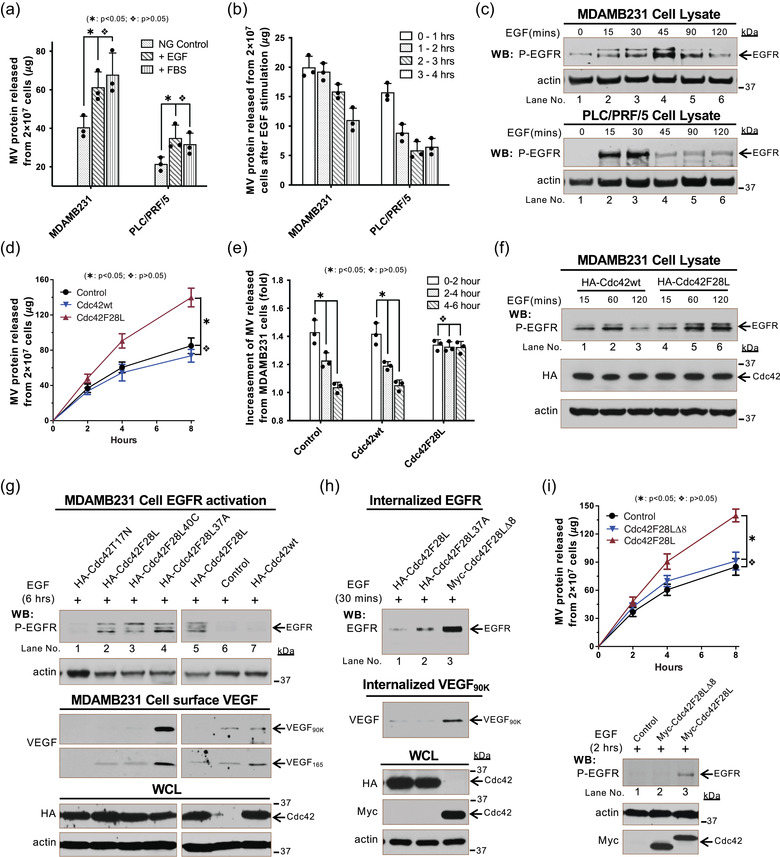
Sustained EGF signaling facilitates MV biogenesis. a, Serum‐starved MDAMB231 or PLC/PRF/5 cells were stimulated by EGF (10 ng/ml) or FBS (10%). MVs shed within 4 h from 2 × 10^7^ these cells were quantified. b, Serum‐starved MDAMB231 or PLC/PRF/5 cells were stimulated by EGF (10 ng/ml). MVs shed within every hour time period (0‐1, 1–2, 2–3, 3–4 h) from 2 × 10^7^ these cells were quantified. c, Lysates (15 μg/samples) of serum‐deprived MDAMB231 (top 2 panels) or PLC/PRF/5 (bottom 2 panels) cells treated with EGF (10 ng/ml) for the indicated lengths of time were immunoblotted with antibodies against phosphorylated‐EGFR and actin. d‐e, MDAMB231 cells transfected with empty vector (Control), HA‐tagged Cdc42wt, or HA‐tagged Cdc42F28L were treated with EGF (10 ng/ml): d, MVs shed from 2 × 10^7^ these cells within 2, 4, and 8 h after EGF‐stimulation were quantified. e, MVs shed from 2 × 10^7^ these cells within every 2‐h time periods (0‐2, 2–4, and 4–6 h) after EGF stimulation were quantified. The folds of the increasement (compared to no EGF stimulation) of MV shedding within every 2‐h time periods after EGF‐stimulation were calculated and plotted. f, Lysates (15 μg/samples) of serum‐deprived MDAMB231 cells expressing HA‐tagged Cdc42wt (lane 1–3) or HA‐tagged Cdc42F28L (lane 4–6) treated with EGF (10 ng/ml) for the indicated lengths of time were immunoblotted with antibodies against phosphorylated‐EGFR, HA, and actin. g, MDAMB231 cells transfected with empty vector (control, lane 6), HA‐tagged T17N (lane 1), HA‐tagged Cdc42F28L (lane 2 and 5), HA‐tagged Cdc42F28L40C (lane 3), HA‐tagged Cdc42F28L37A (lane 4), or HA‐tagged Cdc42wt (lane 7) were treated with EGF (10 ng/ml) for 6 h. Lysates (15 μg/samples) of these cells were immunoblotted with antibodies against phosphorylated‐EGFR and actin (top 2 panels). Proteins on cell surface were labelled with Sulfo‐NHS‐SS‐Biotin and isolated by streptavidin and then analyzed by immunoblot detected VEGF (3rd and 4th panels from top). h, MDAMB231 cells transfected with HA‐tagged Cdc42F28L (lane 1), Cdc42F28L37A (lane 2), or Myc‐tagged Cdc42F28LΔ8 (lane 3), were treated with EGF (10 ng/ml) for 30 min. The internalized cell surface proteins were isolated using Sulfo‐NHS‐SS‐Biotin and MesNa (Sigma) and analyzed by immunoblot detecting EGFR (top panel) and VEGF (2nd panel from top). i, MDAMB231 cells transfected with Myc‐tagged Cdc42F28L or Cdc42F28LΔ8 were treated with EGF (10 ng/ml). MVs shed from 2 × 10^7^ these cells within 2, 4, and 8 h after EGF‐stimulation were isolated and quantified (top plot). Lysates of these cells treated with EGF (10 ng/ml) for 2 h were immunoblotted with antibodies against phosphorylated‐EGFR (top panel), actin and Myc

Cell growth signals have been reported to stimulate MV shedding (Antonyak et al., 2011). Quantification of MVs released from MDAMB231 or PLC/PRF5 cells within 4 h confirmed that EGF or FBS treatment promoted MV shedding (Figure 6a). We further quantified the MVs produced by these cells within every 1‐h time period after EGF stimulation and found that the increased release of MVs from MDAMB231 or PLC/PRF5 cells would continue for about 3 h and 1 h, respectively (Figure 6b). This is approximately consistent with the sustained period of EGFR phosphorylation signalling (Figure 6c).

To determine whether the activity of Cdc42 has an additive effect on EGF‐stimulated MV biogenesis, wild type Cdc42 (Labelled as Cdc42wt hereafter) or the fast cycling Cdc42F28L mutant was expressed in MDAMB231 cells. We quantified the MVs shed from these cells at 2, 4, or 8 h after EGF stimulation and found that Cdc42F28L but not Cdc42wt significantly promoted EGF‐stimulated MV release (Figure 6d). Time course analyses were performed by collecting MVs every 2‐h period (0‐2, 2–4, and 4–6 h) after EGF stimulation and revealed that peak MV shedding by cells expressing Cdc42F28L lasted more than 6 h after EGF stimulation (Figure 6e). Moreover, the elevated MV shedding by Cdc42F28L expression can be inhibited by the EGFR tyrosine kinase inhibitor AG1478 (Figure S5A). The activated fast cycling Cdc42F28L mutant is known to maintain EGFR phosphorylation signalling by blocking EGFR internalization and degradation (Feng et al., 2006; Wu et al., 2003) (Figure 6f, lane 3 *vs* lane 6). Indeed, we found that EGFR phosphorylation in MDAMB231 cells expressing Cdc42F28L is sustained for up to 6 h after EGF stimulation (Figure 6g, top panel, lane 2), which could explain the sustained stimulation of MV release from those cells (Figures 6d and 6e).

To further probe the potential mechanisms of how EGF signalling and Cdc42 regulate the release of VEGF_90K_‐associated MVs, we examined EGFR activation, cell‐surface VEGF_90K_, and the endocytosis of VEGF_90K_ and the EGFR in cells expressing various Cdc42 mutants. When Cdc42F28L, Cdc42F28L37A, or the Cdc42F28L40C mutant were expressed in MDAMB231 cells, EGFR phosphorylation signalling persisted for up to 6 h after EGF stimulation (Figure 6g, top panel, lanes 2–5). In contrast, MDAMB231 cells expressing Cdc42T17N, empty vector (control), or Cdc42wt were unable to maintain EGFR phosphorylation signalling (Figure 6g, top panel, lanes 1, 6, and 7). MDAMB231 cells expressing empty vector (control) or Cdc42wt had small but detectable amounts of VEGF_90K_ and VEGF_165_ on the surface (Figure 6g, 3rd and 4th panels from top, lanes 6 and 7). Cells expressing Cdc42F28L or Cdc42F28L40C had detectable amounts of VEGF_165_, but almost no VEGF_90K_ on the surface (Figure 6g, 3rd and 4th panels from top, lanes 2 and 3). The Cdc42F28L37A‐expressing cells shed the least amount of MVs but had the most VEGF_90K_ on their cell surface (Figure 6g, 3rd and 4th panel from top, lane 4). Of note, these fast cycling Cdc42 mutants had no effect on VEGF trafficking and VEGF_90K_ generation. Together, these data suggest that Cdc42 promotes VEGF_90K_ release by accelerating MV shedding, and that blocking MV shedding traps VEGF_90K_ on the cell surface. The endocytosis of VEGF_90K_ and EGFR after EGF stimulation was examined by assaying the internalization of these proteins. Both Cdc42F28L and Cdc42F28L37A inhibited the internalization of EGFR and VEGF_90K_ (Figure 6h) suggesting that the fast cycling Cdc42F28L mutant maintains the sustained EGFR activation by inhibiting its endocytosis, thereby continuing to stimulate MV biogenesis. Consistently, expression of the Cdc42(F28LΔ8) (Labelled as Cdc42F28LΔ8 hereafter) double mutant that is defective in blocking EGFR endocytosis (Wu et al., 2003) (Cdc42(ΔL8/F28L) in ref. Wu et al., 2003; Figure 6h, top panel, lane 3) did not maintain sustained EGFR signalling (Figure 6i, 3rd panel from bottom) and did not increase MV shedding (Figure 6i).

In addition to promoting VEGF_90K_ release through MV budding, the activated fast cycling Cdc42F28L mutant also inhibited endocytosis of VEGF_90K_ (Figure 6h, 2nd panel from top, lanes 1 and 2). To determine whether Cdc42F28L induced MV shedding depends on both endocytosis inhibition and EGFR signalling, we compared MV shedding by MDAMB231 cells expressing Cdc42F28L or the Cdc42F28LΔ8 mutant in the presence of the EGFR tyrosine kinase inhibitor AG1478. Treatment with AG1478 reduced MV shedding in all tested cells, and cells expressing Cdc42F28L mutant still secreted more MV proteins than cells expressing the Cdc42F28LΔ8 mutant (Figure S5B). When AG1478 treatment blocked EGFR signalling, the main difference between Cdc42F28L and Cdc42F28LΔ8 mutants is the ability to inhibit endocytosis; thus, this result suggests that inhibiting the internalization of cell surface proteins such as VEGF_90K_ also facilitates MV biogenesis. Taken together, these data indicate that both inhibited endocytosis and elevated EGFR signalling are required for Cdc42F28L to promote MV biogenesis.

## 3 DISCUSSION

EVs, which mediate intercellular communication, have attracted significant attention due to their pivotal roles in various physiological and pathophysiological processes, particularly in cancer progression. Small EVs have become the most widely studied EVs and have often been assumed to be the most important (Tkach et al., 2018; Willms et al., 2018).

In fact, MVs may also play extremely important roles in intercellular communication. Small EVs are about 100 nm in diameter and only carry up to a few hundred biomolecules. A simple calculation indicates that even under optimal conditions, for the biomolecules carried by small EVs to be able to modulate cell signalling in a recipient cell, a single recipient cell should simultaneously take up at least tens of thousands of small EVs secreted from tumour cells. This would seem to be unachievable *in vivo* and is a major challenge to the proposed cell signalling mechanism. In fact, it may be a more realistic that small EVs exert functional effects through their miRNA cargo. Compared to small EVs, MVs, on the other hand, have a natural advantage in altering the cellular signalling in the recipient cell *via* protein cargo. They are relatively large, tumour specific, and directly bud off the plasma membrane, therefore naturally carry various signalling proteins originally located on the membrane of MV‐shedding tumour cells. These signalling proteins can easily alter signal transduction on recipient cells.

In this study, we set out to explore the signalling pathways that regulate MV biogenesis. The generation and release of MVs is a complicated dynamic process that is regulated by multiple cellular signals. The heterogeneous nature of MVs, and the technical difficulties in isolating them, are the obstacles that MV studies are facing. It is clear that proper isolation of the entire MV populations, appropriate MV biomarkers, and reliable readouts of MV quantification and cellular function, are necessary for studying signalling pathways that regulate MV biogenesis. In this study, we have shown that a hard spin at 3000 × *g* resulted in a ‘loss’ of the MV(PII) population (MV(PII) presents in sediment together with cell debris after 3000 × *g* spin), which accounts for more than 80% of the MV protein (Figures 1 and S1). We first determined the isolation procedure that would preserve the majority of the MV population. We also found that MVs become saturated in conditioned medium over longer incubation times and will likely mask the differences caused by different treatments. Shorter periods of incubation time are used to avoid saturation. Although VEGF_90K_ is not present on all tumour cell‐derived MVs, it is tumour‐specific and is not present on other EV populations. Therefore, when produced, VEGF_90K_ is a suitable biomarker that also allows for using *in vitro* and *in vivo* angiogenesis assays to study MV functions.

We have demonstrated in this study that the Cdc42‐IQGAP1 signalling pathway regulates MV biogenesis. Cdc42 is a well‐known molecular switch that is activated by upstream signals (e.g., EGF signalling) and then binds downstream effectors to affect many cellular functions. IQGAP1 is one of the Cdc42's downstream effectors and plays an important role in cytoskeletal rearrangement, a necessary process needed for MV biogenesis. Thus, it is not surprising to find the involvement of Cdc42‐IQGAP1 signalling in MV biogenesis. The activated GTP‐GDP exchange cycle, rather than the activated GTP binding state, seems to be critical for Cdc42 to stimulate MV shedding, likely due to the dynamic nature of this process. Here, we should also point out that Cdc42‐IQGAP1 signalling is required, but not sufficient for MV biogenesis. Expression of activated Cdc42 mutants in normal (untransformed) cells does not give them the ability to release MVs. In fact, MV release is regulated by multiple cellular pathways besides the Cdc42‐IQGAP1 signals (Li et al., 2012).

MV formation is a complicated process closely related to actin cytoskeleton rearrangement. Therefore, it is not surprising that many key proteins/signals in the actin cytoskeleton rearrangement are involved in MV biogenesis. The small GTPase Arf6 regulates the MV shedding from LOX cells by promoting myosin light chain (MLC) phosphorylation (Muralidharan‐Chari et al., 2009). However, the overexpression of myosin light chain kinase (MLCK) in Hela cells only caused a moderate increase in MV formation, and the dominantly active Arf6 (Arf6 Q67L) could not promote MV shedding (Li et al., 2012). This indicates that Arf6 does not directly involve in MV biogenesis in some cancer cells. In fact, another GTPase, RhoA, together with ROCK and Lim kinase signals were found to be the key to MV formation in Hela cells (Li et al., 2012). It is not clear whether this is due to the specificity of different cell lines or whether cancer cells rely on different signal transduction pathways under different conditions. The formin homology (FH) protein, DRF3/Dia2, was implicated in the formation of oncosomes, which originate from another type of membrane structure called PM blebs (Charras et al., 2006; Di Vizio et al., 2009; Fackler & Grosse, 2008; Li et al., 2012). PM blebs share some intriguing similarities with MV, raising the possibility that PM blebs and MVs arise through mechanisms partly in common (Li et al., 2012). Interestingly, RhoA.GTP activates the formin protein Dia1. This RhoA‐bound Dia1 associates with IQGAP1 and mediates actin elongation (Watanabe et al., 2015). Arf6/MLC, RhoA/LIMK, and RhoA/Dia1/IQGAP1 all affect the actin elongation. On the other hand, Cdc42, together with IQGAP1 and N‐Wasp, induce actin branching and polymerization (Watanabe et al., 2015). When we assume that both actin elongation and branching are necessary for the formation of actin‐based MVs, we will get a simple ‘picture’ of MV biogenesis, which organizes all these proteins and signal transduction together. Of course, a lot of research on the crosstalk between these proteins/signals is needed to make the image clearer.

In addition to being activated by upstream signals such as EGF signalling and then promoting MV release *via* the Cdc42‐IQGAP1 signalling pathway, Cdc42 also maintains cell surface EGFR by regulating EGFR endocytosis (Wu et al., 2003), providing a sustained signal for MV shedding (Figure 6). This is actually a positive feedback regulatory mechanism. As an important GTPase, Cdc42 has been implicated in the regulation of both clathrin‐dependent (clathrin‐mediated endocytosis, CME) (Giuliani et al., 2009; Watson et al., 2016) and independent (clathrin‐independent endocytosis, CIE) EGFR endocytosis mechanisms (Francis et al., 2015; Howes et al., 2010; Rossatti et al., 2019; Sigismund et al., 2005). We conducted experiments to explore which endocytosis mechanism is the main signalling in our study, and whether EGFR endocytosis directly regulates MV secretion. We found that only CME inhibitors maintained the EGFR phosphorylation in MDAMB231 cells for up to 2 h after 10 ng/ml EGF stimulation (Figure S6A, lane 12; Figure S6B, lane 6 and 9; compared to lane 3 in Figure S6B). This result indicated that in MDAMB231 cells, under 10 ng/ml EGF stimulation, EGFR endocytosis is mainly carried out through CME (Figures S6A, S6B, and S6C). Subsequently, we further examined the release of MV when CME was inhibited. As shown in Figure S6, CME inhibitor increased the release of MV under EGF stimulation (Figures S6D and S6E, histogram 4 *vs* histogram 3). Without EGF stimulation, CME inhibitor had no effect on MV secretion (Figures S6D and S6E, histogram 2 *vs* histogram 1). In addition, under CME inhibitor IKA and EGF stimulation, the dominant negative Cdc42 mutant, Cdc42T17N, still reduced MV release (Figure S6D, histogram 5 *vs* histogram 4). In short, in MDAMB231 cells, CME is involved in the regulation of MV release. However, this regulation is indirect and needs to be achieved by activating Cdc42. The details of how endocytosis affects the MV biogenesis remain to be further studied, which is beyond the scope of this article. Another interesting finding is that in addition to regulating EGF signalling by modulating EGFR endocytosis, Cdc42 also promotes MV biogenesis by regulating the endocytosis of cell surface proteins. This regulation is independent of EGF signalling and its underlying mechanisms are unclear.

**FIGURE 7:**
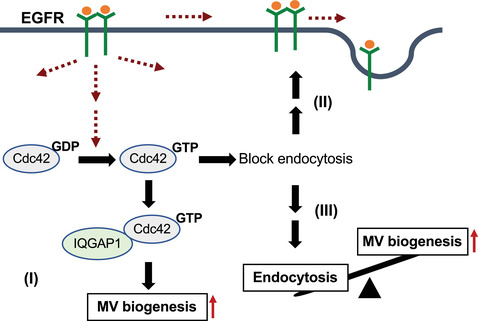
Diagram depicting Cdc42 and IQGAP1 regulate MV biogenesis. Cdc42 activated by upstream signaling (such as EGF) binds to IQGAP1 to facilitate MV shedding (I). Activated Cdc42 maintains EGF signaling by blocking EGFR endocytosis and then help MV biogenesis (positive feedback) (II). Activated Cdc42 also promotes MV biogenesis indirectly by blocking the endocytosis of cell surface proteins (III)

The signalling pathways and mechanisms found in this study that regulate MV biogenesis are summarized in Figure 7. Cdc42 serves as a convergent node for multiple regulatory signals that are required for MV biogenesis. When activated by upstream signalling (such as EGF), Cdc42 binds to IQGAP1 to facilitate MV shedding. Activated Cdc42 maintains EGF signalling by blocking EGFR endocytosis and then helps drive MV biogenesis (positive feedback). Activated Cdc42 also promotes MV biogenesis indirectly by blocking the endocytosis of cell surface proteins. By modulating these signalling pathways, it might be possible to effectively control MV shedding from tumour cells, which could be extremely beneficial for cancer therapy.

## 4 MATERIALS AND METHODS

### 4.1 Reagents

The pan VEGF antibody was obtained from Santa Cruz (Dallas, TX) (SC‐507). The actin, IκBα, and CD‐63 antibodies were from Millipore (Billerica, MA). The flotillin‐2, VEGFR2, and phospho‐VEGFR2 antibodies were from Cell Signalling (Danvers, MA). The IQGAP1, Cdc42, and V5 antibodies were from Invitrogen (Waltham, MA). The HA, and Myc antibodies were from Convance. Brefeldin A and sodium 2‐mercaptoethanesulphonate was from Sigma (St. Louis, MO). CME inhibitor Ikarugamycin (IKA) was from MedChemExpress (MCE) (Monmouth Junction, NJ). CME inhibitors Pitstop 1 and Pitstop 2 were from Abcam (Cambridge, UK). CIE inhibitor Filipin III was from APExBIO (Houston, TX). The Steriflip PVDF filters (0.1 or 0.22 μm pore size) and centrifugal filters, were from Millipore.

RNAis against IQGAP1 and Cdc42 were purchased from Invitrogen (Waltham, MA, USA). Plasmids encoding Cdc42Q61L, Cdc42F28L, T17N, Cdc42F28L37A, Cdc42(F28L40C, Cdc42F28LΔ8 (Cdc42(ΔL8/F28L) in ref. Wu et al., 2003), IQGAP1, IQGAP1Δ (IQGAP1‐FΔMK in ref. Rittmeyer et al., 2008) were generated and characterized by former members in Cerione laboratory (Li et al., 1999; Lin et al., 1997; Rittmeyer et al., 2008; Wang et al., 2009; Wu et al., 2003).

### 4.2 Cell Culture and transfections

Cell Culture. The MDAMB231, U87, and MCF10A cell lines were grown in RPMI‐1640 medium containing 10% FBS. The PLC/PRF/5 and NIH 3T3 cell lines were grown in DMEM containing 10% FBS. The HUVEC cell lines were grown in EGM medium (Lonza) containing Endothelial Cell Growth Medium Bullet Kit. Expression constructs were transfected into cells using Lipofectamine. Control and RNAis were introduced into cells using Lipofectamine RNAiMAX reagent (Life technologies, USA). As indicated, cells were incubated with serum‐free medium containing combinations of 0.01 μg/ml EGF, 10μM BFA, and 4μM AG1478.

### 4.3 Isolation of MVs

Conditioned medium from at least 2 × 10^7^ serum‐starved various types of cells was collected and partially clarified by three consecutive centrifugations at 1000 rpm (∼300 × *g*) for 5 min, or by filtration using a Millipore ultrafree PVDF filter with a 3.1 μm pore size. The partially clarified conditioned medium was filtered using a Millipore ultrafree PVDF filter with a 0.1 μm or 0.22 μm pore size. The MVs retained by the PVDF membrane were resuspended in serum‐free medium. The size and purity of MVs were then examined with fluorescent staining after isolation. To generate MV lysates, The MVs retained by the PVDF membrane were washed with PBS for 3 times (3 × 5 ml PBS, do not let MVs dry on the membrane) and then lysed in cell lysis buffer. The protein concentrations of the MV preparations were determined using the Bio‐Rad DC protein assay.

To isolate MV(PI) and MV(PII), the MVs retained by the PVDF membrane were washed with PBS for 3 times (3 × 5 ml PBS, do not let MVs dry on the membrane) and then resuspended in serum‐free medium and then centrifugated at 3,000 × *g* for 15 min (MV(PII)). The supernatant was further centrifugated at 12,000 × *g* for 45 min (MV(PI)) (Figure S1C).

### 4.4 Isolation of small EVs

Conditioned medium from at least 2 × 10^7^ serum‐starved various types of cells were collected and partially clarified by three consecutive centrifugations at 1000 rpm (∼300 × *g*) for 5 min, or by filtration using a Millipore ultrafree PVDF filter with a 3.1 μm pore size. The supernatant was then centrifugated at 3000 × *g* (3 × 15 min), 12,000 × *g* (3 × 30 min), and followed by 120,000 × *g* for 4 h. The pellet was gently washed with PBS for 3 times and then the isolated small EVs were lysed or resuspended in PBS (Figure S1D).

**Light scattering**. The size and purity of MVs isolated from conditioned medium were measured using the Nano Zetasizer (Malvern) at the Nanobiotechnology Center, Cornell University or Core Facility Center for life Sciences, University of Science and Technology of China.

### 4.5 Nanoparticle tracking analysis (NTA)

The quantification and purity of MVs isolated from conditioned medium were measured using the ZetaView BASIC NTA (Particle Metrix) at Cancer Research Center, University of Science and Technology of China.

### 4.6 Proteomic analysis

Proteomic analysis of isolated MV preparations was performed at the Nanobiotechnology Center, Cornell University or Core Facility Center for life Sciences, University of Science and Technology of China. Data were analyzed and presented by software SangerBox (http://sangerbox.com) and TBtools (https://github.com/CJ-Chen/TBtools).

### 4.7 *In vivo* angiogenesis assays

The Directed*in vivo* Angiogenesis Assay Kit (Trevigen, Gaithersburg, MD) was used to assay endothelial cell recruitment in mice according to the manufacturer's protocol. Briefly, implant grade silicone cylinders that are closed at one end (angioreactors) were filled with 20 μl of BME gel premixed with 2 μl of RPMI (vehicle control), or with or without various activators such as MVs and small EVs. The angioreactors were then implanted subcutaneously in the dorsal flanks of nude mice. One week later, the angioreactors were removed. The vascular endothelial cells that migrated (invaded) into the angioreactors were quantified using the FITC‐Lectin Detection protocol (Napoli et al., 2011).

### 4.8 Tubulogenesis assays

The *in vitro* Angiogenesis Assay Tube Formation Kit (Trevigen) was used according to the manufacturer's instructions. Briefly, BME solution was added into 96‐well plates (50 μl per well), and the plates were incubated at 37°C for 60 min. HUVECs were diluted with Endothelial Basal Medium in the presence or absence of various activators such as MVs and small EVs. The HUVECs (1 × 10^4^ cells) were then added to each well containing the gelled BME. The plates were incubated at 37°C in a CO_2_ incubator for 4 h, at which time the HUVEC‐formed tubular networks were visualized using a light microscope.

### 4.9 Cell surface protein biotinylation assay

Cell surface protein was isolated using Pierce Cell Surface Protein Isolation Kit (Pierce, Rockford, IL, USA). Briefly, MDAMB231 cells expressing various mutants were washed three and suspended in ice‐cold PBS (pH 8.0). Biotinylation was performed by incubating cells in PBS containing EZ‐link Sulfo–NHS–SS–biotin for 30 min. Cells were subsequently washed 3 times with ice‐cold PBS (pH 8.0) to remove unreacted reagent. Labelled cells were lysed and cell surface proteins were immunoprecipitated with avidin resin and analyzed by immunoblot with VEGF antibody.

### 4.10 Cell surface protein internalization assay

Cell surface protein internalization was performed using Pierce Cell Surface Protein Isolation Kit as well (Ghossoub et al., 2014). Briefly, MDAMB231 cells expressing various mutants were washed 3 times with PBS+MgCl_2_ and biotinylated for 30 min with reducible Sulfo‐NHS‐SS‐biotin at 4°C. After stimulation with EGF 10 ng/ml, cell surface biotin was removed by treatment (2 × 20 min) at 4°C with sodium 2‐mercaptoethanesulphonate (MesNa). The internalized proteins were immunoprecipitated with avidin resin and analyzed by immunoblot with EGFR and VEGF antibodies.

### 4.11 Immunofluorescence

Cells were fixed with 3.7% paraformaldehyde and then permeabilized with PBS containing 0.1% Triton X‐100. Samples were incubated with the primary antibodies and then with Oregon green 488‐conjugated secondary antibodies. The lipid‐binding membrane dye, FM1‐43X, was used to stain MV and plasma membrane, Rhodamine‐conjugated phalloidin was used to stain (F)‐actin filaments, and DAPI was used to label nuclei. Fluorescent microscopy images were captured and processed using IPLab software (Scientific Instrument Company, Inc., Campbell, CA, USA).

### 4.12 Confocal microscopy

Cells were fixed in 4% paraformaldehyde in PBS for 15 min and stained for the indicated proteins. Images were obtained with a Zeiss LSM 510 Meta confocal microscope (Carl Zeiss, Germany) using a Plan‐Apochromat 63 × /1.40 oil objective. The pinhole size was set to 1 airy unit for all channels.

### 4.13 Data analyses

For MV and small EV quantification, and NTA: The data shown represents the mean ± SD from at least three independent experiments. For tubulogenesis assays: Photographs of the cell cultures were taken and the lengths of the tubes that formed for each condition were determined using ImageJ (NIH). The data shown represents the mean ± SD from at least three independent experiments. For *in vivo* angiogenesis assays: The data were expressed in Relative Fluorescent Units (RFUs). Relative invasion = Test sample (RFU) / Negative Control (RFU). The data shown represent the mean ± SD from 3–6 angioreactors.

All *P* values were determined by the student's *t*‐test and *P* < 0.05 is considered as significant difference.

## AUTHOR CONTRIBUTIONS

Qiyu Feng and Hongyang Wang conceived the study. Qiyu Feng wrote the manuscript. Qiyu Feng, Jing Wang, Xiangjin Zhuang, and Ha Si performed the majority of the experiments. Kai Su Greene, Marc A. Antonyak, and Kristin F. Wilson performed the light scattering analysis and the Proteomic screening experiment. Joseph E. Druso contributed to the mouse experiments. Richard A. Cerione helped with data analysis and manuscript preparation.

## CONFLICT OF INTEREST

The authors declare no conflict of interest.

## Supporting information



Supplementary information
