# Supplementary material for: Cdc42 functions as a regulatory node for tumour‐derived microvesicle biogenesis
Source: J Extracell Vesicles. 2021 Jan 12;10(3):e12051. doi: 10.1002/jev2.12051 (PMC7804048; doi:10.1002/jev2.12051)
Supplement: Supplementary file 1 — Supplementary information [file JEV2-10-e12051-s001.pdf]

Figure S1

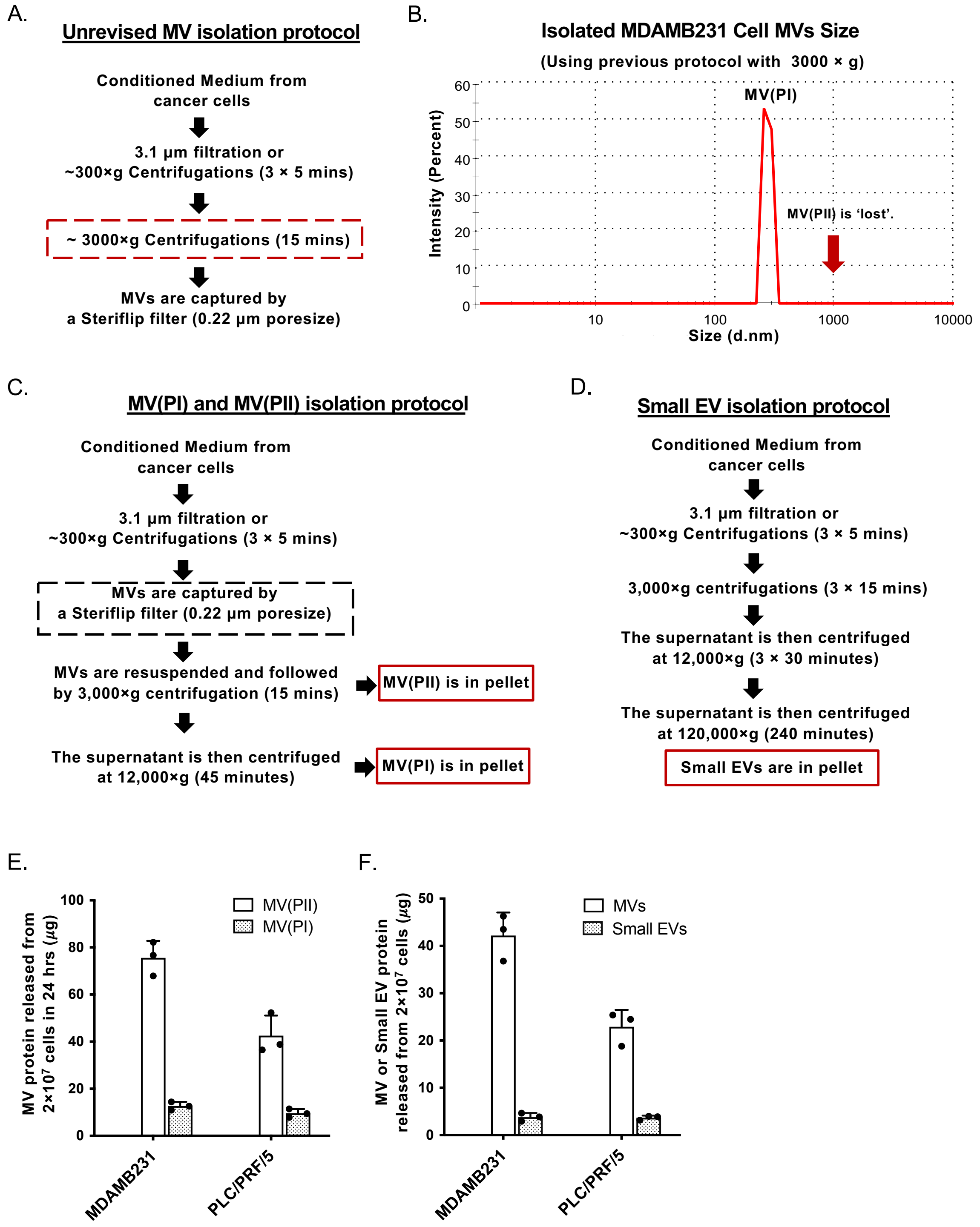
A.
Unrevised MV isolation protocol
Conditioned Medium from cancer cells
3.1 µm filtration or ~300×g Centrifugations (3 × 5 mins)
~ 3000×g Centrifugations (15 mins)
MVs are captured by a Steriflip filter (0.22 µm poresize)
B.
Isolated MDAMB231 Cell MVs Size
(Using previous protocol with 3000 × g)
MV(PI)
MV(PII) is 'lost'.
Intensity (Percent)
Size (d.nm)
0
10
20
30
40
50
60
10
100
1000
10000
C.
MV(PI) and MV(PII) isolation protocol
Conditioned Medium from cancer cells
3.1 µm filtration or ~300×g Centrifugations (3 × 5 mins)
MVs are captured by a Steriflip filter (0.22 µm poresize)
MVs are resuspended and followed by 3,000×g centrifugation (15 mins)
MV(PII) is in pellet
The supernatant is then centrifuged at 12,000×g (45 minutes)
MV(PI) is in pellet
D.
Small EV isolation protocol
Conditioned Medium from cancer cells
3.1 µm filtration or ~300×g Centrifugations (3 × 5 mins)
3,000×g centrifugations (3 × 15 mins)
The supernatant is then centrifuged at 12,000×g (3 × 30 minutes)
The supernatant is then centrifuged at 120,000×g (240 minutes)
Small EVs are in pellet
E.
MV protein released from 2×10^7 cells in 24 hrs (µg)
MV(PII)
MV(PI)
0
20
40
60
80
100
MDAMB231
PLC/PRF/5
F.
MV or Small EV protein released from 2×10^7 cells (µg)
MVs
Small EVs
0
10
20
30
40
50
MDAMB231
PLC/PRF/5

Figure S1 (Supplementary Figure for Figure 1). Isolate and quantify tumor cell-derived MVs. **A,** Unrevised MV isolation protocol has steps of ~3000×g centrifugations. **B,** MVs isolated from conditioned medium collected from MDAMB231 cells using unrevised protocol (has steps of ~3000×g centrifugations) was analyzed by dynamic light scattering. The MV population II(MV(PII)) was totally 'lost' (indicated by arrow). **C,** MV population I and II (MV(PI) and MV(PII)) isolation protocol. **D,** Small EV isolation protocol. **E,** MV population I and II (MV(PI) and MV(PII)) produced within 24 hours by 2×10^7 MDAMB231 or PLC/PRF/5 cells were quantified. MV(PII) accounted for more than 80% of total MV protein. **F,** MVs or small EVs produced within 4 hours by 2×10^7 MDAMB231 or PLC/PRF/5 cells were quantified. MV protein was at least 10 times more than small EV protein.

Figure S2

A.

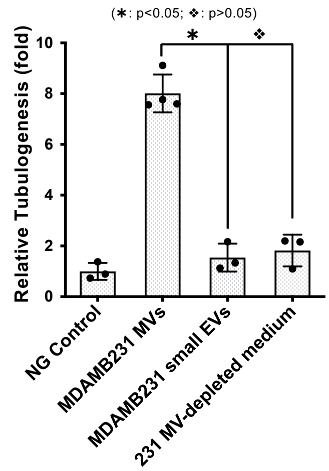

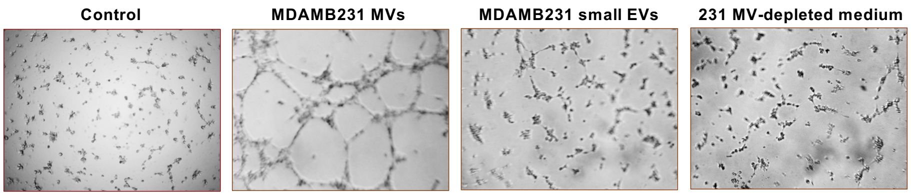

Relative stimulation of Tubulogenesis

Samples	NG Control	MDAMB231 MVs	MDAMB231 small EVs	231 MV-depleted medium
Relative fold	1.000 ± 0.333	8.01 ± 0.742	1.54 ± 0.551	1.82 ± 0.624

B.

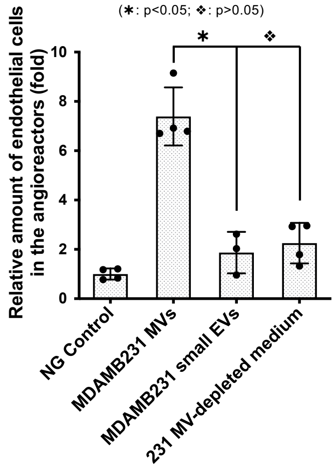

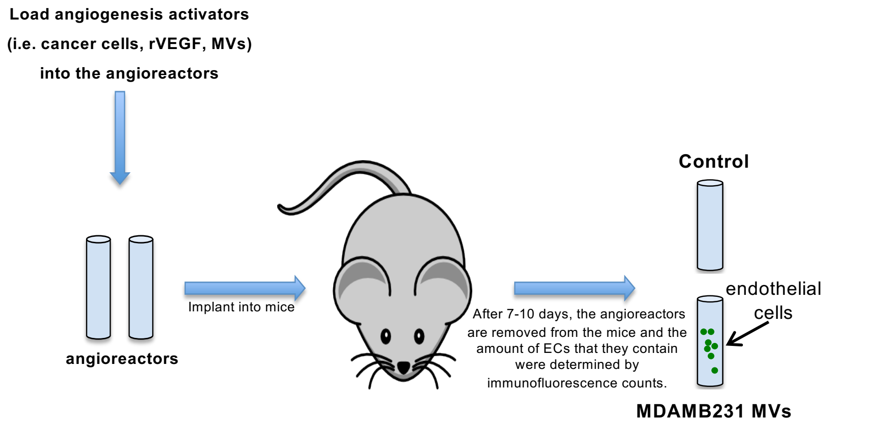

(from Fig. 2a in ref. #20 (Feng et al.))

Amounts of endothelial cells in the angioreactors

Samples	NG Control	MDAMB231 MVs	MDAMB231 small EVs	231 MV-depleted medium
Avg. IF counts	1785 ± 399.7	13193 ± 2106	3338 ± 1499	4016 ± 1464
Relative fold	1.000 ± 0.224	7.391 ± 1.18	1.87 ± 0.84	2.25 ± 0.82

Figure S2 (Supplementary Figure for Figure 2). **A,** Data and images of the tubulogenesis assays in Figure 2E was presented. **B,** Schematic of the *in vivo* angiogenesis assay (also in ref. #20, Figure 2a). Angioreactors are loaded with growth factor reduced basement membrane extract (BME, Trevigen) and either medium alone (control), or with MDAMB231 cell MVs, and then implanted into mice. Seven-ten days later, they are removed and the relative amounts of endothelial cells that entered (invaded) the reactors are determined. Data of the *in vivo* angiogenesis assay in Figure 2F was presented.

Figure S3

A.

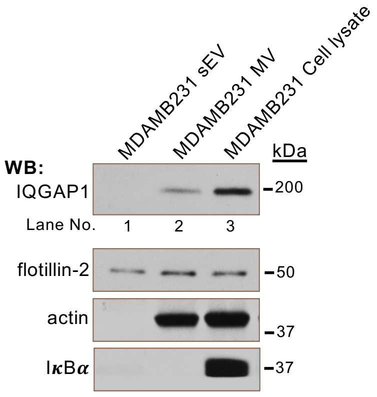

MDAMB231 sEV
MDAMB231 MV
MDAMB231 Cell lysate
kDa
WB:
IQGAP1
200
Lane No. 1 2 3
flotillin-2
50
actin
37
IκBα
37

B.

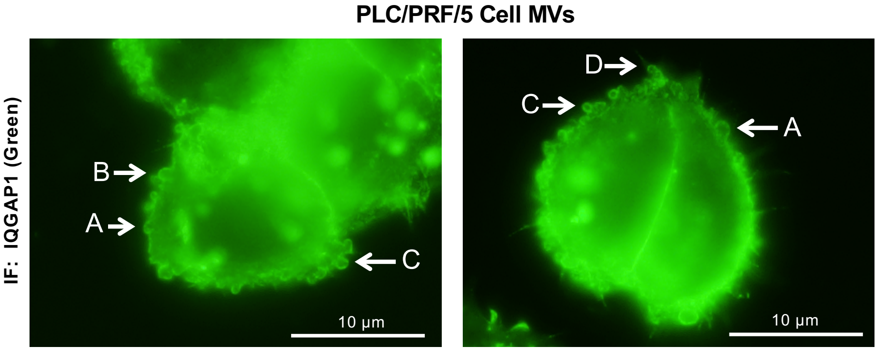

PLC/PRF/5 Cell MVs
IF: IQGAP1 (Green)
B
A
C
10 μm
D
C
A
10 μm

Figure S3 (Supplementary Figure for Figure 3). A, Small EVs (lane 1) or MVs (lane 2) shed from MDAMB231 cells and MDAMB231 cell lysate (lane 3, 10μg/samples) were immunoblotted with antibodies against IQGAP1, the MV marker protein flotillin-2, actin, and the cytosolic-specific marker IκBα. **B,** Serum-starved PLC/PRF/5 cells were analyzed by immunofluorescent microscopy using IQGAP1 antibody. Arrows indicate the MVs at different steps of shedding: budding (arrow A), stretching (arrow B), closing (arrow C), and release (arrow D).

Figure S4

A.

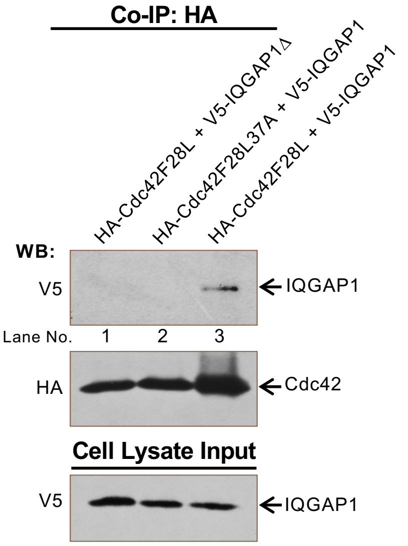

B.

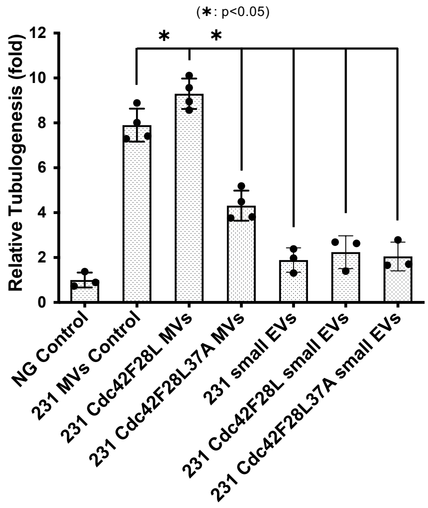

Relative stimulation of Tubulogenesis

Samples	NG Control	231 MV Control	231 Cdc42F28L MVs	231 Cdc42F28L37A MVs
Relative fold	1.000 ± 0.333	7.90 ± 0.731	9.30 ± 0.679	4.31 ± 0.670
Samples		231 small EVs	231 Cdc42F28L small EVs	231 Cdc42F28L37A small EVs
Relative fold		1.890 ± 0.542	2.24 ± 0.73	2.05 ± 0.64

C.

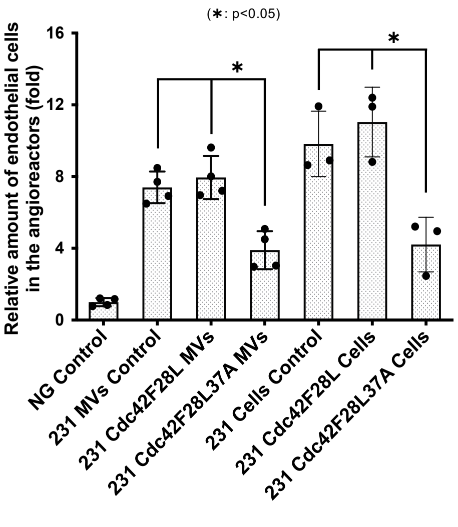

Amounts of endothelial cells in the angioreactors

Samples	NG Control	231 MV Control	231 Cdc42F28L MVs	231 Cdc42F28L37A MVs
Avg. IF counts	1785 ± 399.7	13209 ± 1571	14191 ± 2142	6962 ± 1892
Relative fold	1.000 ± 0.224	7.4 ± 0.880	7.95 ± 1.200	3.9 ± 1.060
Samples		231 Cells Control	231 Cdc42F28L Cells	231 Cdc42F28L37A Cells
Avg. IF counts		17529 ± 3249	19706 ± 3463	7515 ± 2713
Relative fold		9.82 ± 1.820	11.04 ± 1.940	4.21 ± 1.520

Figure S4 (Supplementary Figure for Figure 5). A, MDAMB231 cells expressing HA-tagged Cdc42F28L together with V5-tagged IQGAP1Δ (lane 1), HA-tagged Cdc42F28L37A together with V5-tagged wild type IQGAP1 (lane 2), HA-tagged Cdc42F28L together with V5-tagged wild type IQGAP1 (control, lane 3) were lysed. Immunoprecipitations (IPs) using a HA antibody were performed on the cell lysates (200 μg protein). The immunocomplexes were blotted with V5 and HA antibodies. The cell lysates were probed with an V5 antibody to confirm equivalent amounts of sample were used in each IP (bottom panel). **B,** Data of the tubulogenesis assays in Figure 5E was presented. **C,** Data of the *in vivo* angiogenesis assay in Figure 5F was presented.

Figure S5

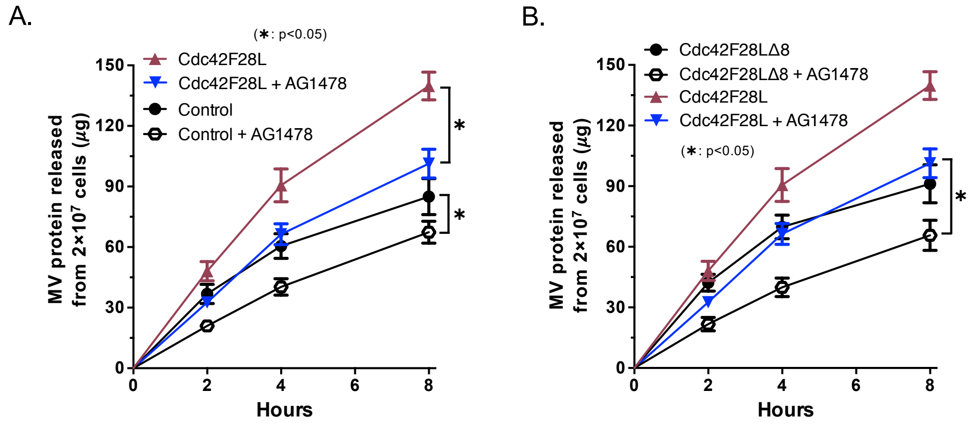

A.
(✱: p<0.05)
Cdc42F28L
Cdc42F28L + AG1478
Control
Control + AG1478
MV protein released from 2×10^7 cells (μg)
150
120
90
60
30
0
0
2
4
6
8
Hours
*
*
B.
Cdc42F28LΔ8
Cdc42F28LΔ8 + AG1478
Cdc42F28L
Cdc42F28L + AG1478
(✱: p<0.05)
MV protein released from 2×10^7 cells (μg)
Hours
*

Figure S5 (Supplementary Figure for Figure 6). A, MDAMB231 cells expressing empty vector (Figure 6D) without (control) or with AG1478 treatment, or HA-tagged Cdc42F28L (Figure 6D) without or with AG1478 treatment were stimulated with EGF (10ng/ml). MVs shed from 2×10^7 these cells within 2, 4, and 8 hours after EGF-stimulation were quantified. The difference of the MV shed from these cells with or without AG1478 treatment was statistically significant (✱: $p<0.05$). **B,** MDAMB231 cells expressing Myc-tagged Cdc42F28L (Figure 6I) without (control) or with AG1478 treatment, or Myc-tagged Cdc42F28LΔ8 (Figure 6I) without or with AG1478 treatment were stimulated with EGF (10ng/ml). MVs shed from 2×10^7 these cells within 2, 4, and 8 hours after EGF-stimulation were quantified. The difference of the MV shed from these cells with or without AG1478 treatment was statistically significant (✱: $p<0.05$).

Figure S6

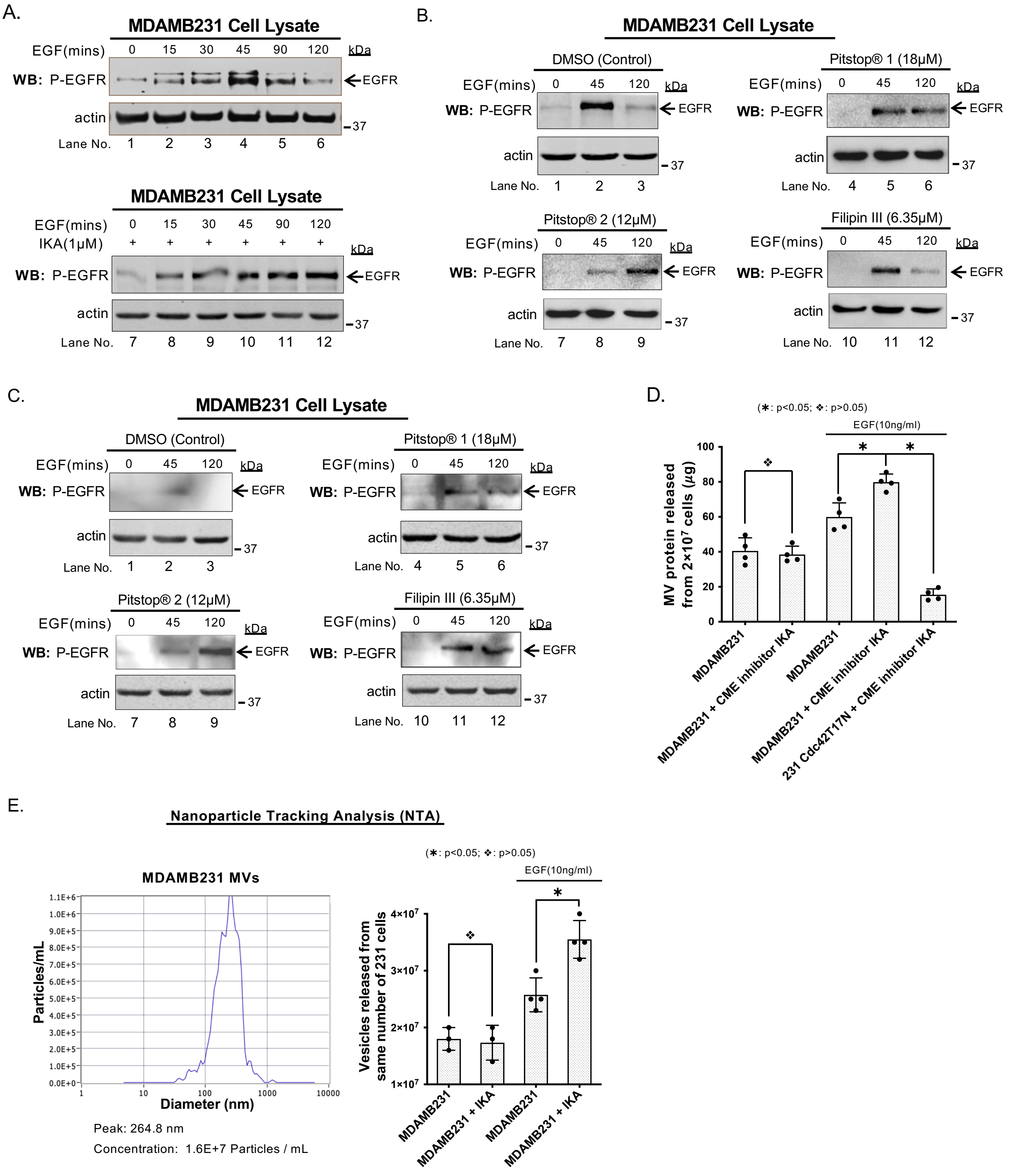

A.
MDAMB231 Cell Lysate
EGF(mins) 0 15 30 45 90 120
kDa
WB: P-EGFR
EGFR
actin
37
Lane No. 1 2 3 4 5 6
MDAMB231 Cell Lysate
EGF(mins) 0 15 30 45 90 120
IKA(1μM) + + + + + +
WB: P-EGFR
actin
Lane No. 7 8 9 10 11 12
B.
MDAMB231 Cell Lysate
DMSO (Control)
Pitstop® 1 (18μM)
Pitstop® 2 (12μM)
Filipin III (6.35μM)
EGF(mins) 0 45 120
Lane No. 1 2 3
Lane No. 4 5 6
Lane No. 7 8 9
Lane No. 10 11 12
C.
MDAMB231 Cell Lysate
D.
(✱: p<0.05; ❖: p>0.05)
EGF(10ng/ml)
MV protein released from 2×10^7 cells (μg)
MDAMB231
MDAMB231 + CME inhibitor IKA
MDAMB231
MDAMB231 + CME inhibitor IKA
231 Cdc42T17N + CME inhibitor IKA
E.
Nanoparticle Tracking Analysis (NTA)
MDAMB231 MVs
Particles/mL
Diameter (nm)
Peak: 264.8 nm
Concentration: 1.6E+7 Particles / mL
(✱: p<0.05; ❖: p>0.05)
EGF(10ng/ml)
Vesicles released from same number of 231 cells
MDAMB231
MDAMB231 + IKA
MDAMB231
MDAMB231 + IKA

Figure S6 (Supplementary Figure for Figure 7). **A,** Top two panels: (same as Figure 6C, top two panels); Bottom two panels: Lysate (15μg/samples) of serum-deprived MDAMB231 cells (pre-incubated with CME inhibitor Ikarugamycin (IKA, 1μM) for 30 minutes) treated with EGF (10ng/ml) for the indicated lengths of time were immunoblotted with antibodies against phosphorylated-EGFR and actin. **B,** Membrane fraction lysate (15μg/samples) of serum-deprived MDAMB231 cell (pre-incubated with 0.5% DMSO (control), CME inhibitor Pitstop® 1 (18μM), CME inhibitor Pitstop® 2 (12μM), or CIE inhibitor Filipin III (6.35μM) for 30 minutes) treated with EGF (10ng/ml) for the indicated lengths of time were immunoblotted with antibodies against phosphorylated-EGFR, and actin. **C,** Membrane fraction lysate (15μg/samples) of serum-deprived MDAMB231 cell (pre-incubated with 0.5% DMSO (control), CME inhibitor Pitstop® 1 (18μM), CME inhibitor Pitstop® 2 (12μM), or CIE inhibitor Filipin III (6.35μM) for 30 minutes) treated with EGF (1ng/ml) for the indicated lengths of time were immunoblotted with antibodies against phosphorylated-EGFR and actin. **D,** MDAMB231 cell expressing empty vector (histogram 1-4) or HA-tagged Cdc42T17N (histogram 5) were pre-incubated with (histogram 2, 4, and 5) or without CME inhibitor IKA (1μM) (histogram 1 and 3) for 30 minutes and then stimulated with (histogram 3-5) or without EGF (10ng/ml) (histogram 1 and 2). MVs released within 4 hours from 2×10^7 these cells were isolated and quantified. With EGF stimulation, CME inhibitor IKA promoted MV release (histogram 4 *vs* histogram 3; ✱: $p<0.05$) and Cdc42T17N still inhibited MV release (histogram 5 *vs* histogram 4; ✱: $p<0.05$). Without EGF stimulation, CME inhibitor IKA did not increase MV release (histogram 2 *vs* histogram 1; ❖: $p>0.05$). **E,** MDAMB231 cells were pre-incubated with (histogram 2 and 4) or without CME inhibitor IKA (1μM) (histogram 1 and 3) for 30 minutes and then stimulated with (histogram 3 and 4) or without EGF (10ng/ml) (histogram 1 and 2). MVs released within 4 hours from same number of these cells were isolated and then quantified by Nanoparticle tracking analysis (NTA). Left panel: A example of the vesicle number counting result (MDAMB231 MV) was shown. Right panel: A plot presented the number of vesicles measured by NTA. With EGF stimulation, CME inhibitor IKA (1μM) promoted MV release (histogram 4 *vs* histogram 3; ✱: $p<0.05$). Without EGF stimulation, CME inhibitor IKA did not increase MV release (histogram 2 *vs* histogram 1; ❖: $p>0.05$).
